# Aging hampers neutrophil extracellular traps (NETs) efficacy

**DOI:** 10.1007/s40520-022-02201-0

**Published:** 2022-08-03

**Authors:** Maurizio Sabbatini, Elisa Bona, Giorgia Novello, Mario Migliario, Filippo Renò

**Affiliations:** 1 Department of Sciences and Innovative Technology, Università del Piemonte Orientale, Viale T. Michel 11, 15121 Alessandria, Italy; 2 Department of Sciences and Innovative Technology, Università del Piemonte Orientale, Piazza San Eusebio 5, 13100 Vercelli, Italy; 3 Department of Health Sciences and Innovative Research Laboratory for Wound Healing, Università del Piemonte Orientale, via Solaroli, 17, 28100 Novara, Italy

**Keywords:** Aging, NETosis, LPS, HaCaT, DNA fragmentation

## Abstract

**Background:**

NETosis is a neutrophil-mediated defense mechanism during which DNA and enzymes are extruded forming a network (NETs) trapping and killing different pathogens. NETosis is reduced in both mice and humans during aging.

**Aims:**

We explored the difference in the efficacy of NETs released in elderly (> 65 years) versus adults (20–50 years) subjects in inhibiting *Staphylococcus aureus* growth and activating the growth of keratinocytes.

**Methods:**

Neutrophil granulocytes, obtained from venous blood both in healthy elderly and adult subjects, were stimulated by LPS (0–250 µg/ml) to induce the formation of NET. NETs were quantified by SYBR Green staining and growth inhibition of *S. aureus* was evaluated by disk diffusion test. Furthermore, NETs (0–500 ng/ml) were added to immortalized human keratinocytes (HaCaT cells), and their proliferation was evaluated by MTT assay after 24 h. Finally, the DNA size of NETs was evaluated by flow cytometry after SYBR Green staining.

**Results:**

Greater production of NETs was observed in elderly subjects than in adults, but these NETs showed reduced bactericidal capacity and HaCaT cells’ proliferation stimulation. The activities of the NETs are related to the size of the extruded DNA threads, and when NETs size was analyzed, DNA from elderly showed a higher size compared to that obtained by adults.

**Discussion:**

Unexpected results showed aging-related NETs structural modification resulting in both a lower antimicrobial activity and keratinocyte proliferation stimulation compared to NETs obtained from adults.

**Conclusions:**

The NETs DNA size observed in elderly subjects has not been previously reported and could be part of other pathogenic mechanisms observed in aging.

## Introduction

Neutrophil granulocytes play a crucial role in the innate immune defense against bacteria, fungi and virus and they are potentially harmful to the host as well.

Their activation and microbicidal activities are strictly controlled by a plethora of stimuli, and recent evidence suggests that they are quite versatile and can perform previously unsuspected functions, such as reverse transmigration, crosstalk and regulation of other leukocyte populations [1].

The antimicrobial and cytotoxic action mechanisms of neutrophils consist in phagocytosis, in the generation of reactive oxygen species (ROS), and in the degranulation of several microbicidal factors (α-defensins, cathelicidin, elastase, cathepsin G and lactoferrin).

An additional antimicrobial action of neutrophil granulocytes was reported by Brinkmann and colleagues [2] who observed the extrusion by neutrophils of a meshwork of chromatin fibers decorated with granule-derived antimicrobial peptides, and enzymes capable to kill Gram-positive and Gram-negative bacteria. This defensive meshwork has received the denomination of neutrophil extracellular traps (NETs). NETs are composed of highly decondensed chromatin fibers having diameter from 15 to 17 nm derived from nuclear components accompanied by histone proteins complexed with microbicidal globular proteins, such as elastase, cathepsin G, myeloperoxidase, normally stored in neutrophil granules [2, 3].

NETs are released into the extracellular space where the chromatin meshwork traps microbes limiting their diffusion and collecting the neutrophil factors, thus increasing the microbicidal effects [4]. NETs release becomes fundamental as a defense mechanism when the size of the pathogens makes phagocytosis an unreliable process [5].

A further ability with which NETs carry out their microbicidal activity is related to the ability of DNA to induce the chelation of manganese and other ions. As a consequence of the chelating activity of the DNA, present in the NETs meshwork, the altered transport of the ions prevents the survival of microbes [6, 7]. Furthermore, it has been observed that NETs do not have an active function in eliminating pathogens only, but also, they regulate the local inflammatory process [8, 9]. Furthermore, the importance of NETs structure in fighting microbial invasion has been demonstrated in mice infected with pathogens which showed greater bacterial dissemination when treated with exogenous DNases [5, 10].

In summary, NETs act as a complex defence system using DNA threads, microbicidal enzymes trapped in the DNA mesh and human complement system [11] and by enhancing the activity of bystander T cells [12]. Furthermore, NETs help the tissue regeneration process, particularly in wound healing, by stimulating keratinocytes proliferation [13].

This complex modularity of NETs explains why their qualitative or quantitative alteration could cause pathologies or aggravate organ damage in several pathologies [14].

During aging, immune performance is reduced and neutrophils activity is also impaired, increasing susceptibility to microbial invasion [15]. In particular, in elderly mice NETs production induced by both PMA and *S. aureus* was strongly reduced [16], as well as in human neutrophils from elderly subjects primed with TNF-α and stimulated with LPS [17]. However, the production of NETs in healthy elderly human subjects has not been extensively studied, especially in non-preconditioned cells.

Likewise, no information about the length of DNA present in NETs was produced; on the other hand, particular attention was paid to the dimension and structure of NET meshwork [2]. LPS stimulation activates the TLR4-JNK pathway that is critical in determining the NETotic fate of neutrophils in response to bacterial invasion [18]. Furthermore, TLR4 is a cell membrane receptor widely present on the cell that has been shown to be involved in wound healing by affecting keratinocytes’ migration [19].

In this paper, to clarify the effect of aging on NET activity, we measured NETs production in neutrophils obtained from adult and elderly subjects after physiological stimulation with LPS and tested their antimicrobial capacity against *S. aureus* and their healing ability in stimulating the growth of human keratinocytes.

## Materials and methods

### Granulocyte isolation

Human blood from healthy volunteer donors was used with prior informed consent and after receiving the approval of the Hospital “Maggiore della Carità” Ethic Committee (C.E. 61/10).

Donors were divided into two groups named adult (10 subjects, range 20–50 years, mean ± S.D. = 35 ± 10.9) and the elderly (8 subjects over 65 years of age, range 71–75, mean ± S.D. = 71.8 ± 1.9).

Venous blood samples were collected in Vacutainer tubes with lithium-heparin anticoagulants and were processed to separate blood to its various cellular components (erythrocytes, leukocytes).

Initially, the blood was diluted 1:2 with PBS and subsequently stratified on Lymphocyte (PromoCell, Heidelberg, Germany) previously dispensed in falcon tubes.

The three upper phases were discarded, whereas the part with erythrocytes and granulocytes was added with ammonium chloride solution (150 mM NH_4_Cl, 10 mM NaHCO_3_, 1 mM EDTA, pH = 7.4) and conserved in ice for 20 min to isolate granulocytes from erythrocytes.

Once the sample was lysed, centrifugation at 800 rpm (120 × *g*) was performed for 5 min to obtain a pellet at the bottom of the tube containing granulocytes.

At the end, they were suspended in RPMI at 10% of FBS (Euroclone, Milan, Italy) at 10^6^ cells/ml concentration.

### NETs fluorescence microscopy visualization

NETosis is identified by the production of neutrophil extracellular traps (NETs) formed by a network of DNA and mainly histone proteins. To assess the presence of NETs using fluorescence microscopy visualization, 1 × 10^5^ neutrophils obtained from the two experimental groups, were seeded on 24 multi-wells and treated with LPS 250 μg/ml for 4 h to induce NETs production (positive control). At the end of the experiments, cells were fixed overnight at 4 °C in the dark using a 3.7% formaldehyde/3% sucrose solution in PBS and stained with SYBR Green dye (10 μg/ml) (Thermo Fisher Scientific, Waltham, Massachusetts, USA). Representative images were then acquired using a fluorescence microscope (Leica, Italy), and analyzed with Image J software at an original magnification of × 40.

### NETs quantification

NETs were produced according to the literature procedure [20]. Briefly, 1 × 10^5^ neutrophils/well were seeded into a 96 multi-well culture plate in Hanks’ Balanced Salt solution (HBSS) and stimulated with different LPS concentrations (0–250 μg/ml, to activate NETs formation), Triton X-100 (to evaluate total DNA content) or left untreated (negative control and DNase digested samples). After 2 h, 5 U DNase was added to all wells (excluding controls and Triton X-100 treated samples) and 45 min after DNase addition, cell-impermeable fluorescent DNA dye Sytox Green (Thermo Fisher Scientific, Waltham, MA, USA) was added to each well and the plate was incubated for further 15 min. At the end of the incubation, Sytox Green fluorescence (λex504 nm/λem523 nm) was quantified using a microplate reader (Victor X4, Perkin-Elmer, Waltham, MA, USA). Extracellular DNA content was expressed as percentage of total DNA obtained by subtracting the fluorescence intensity of the DNase containing wells from the comparative control and dividing the obtained result by the fluorescence intensity of Triton X-100 containing wells as indicated by the following formula:

$$\% {\text{extracellular DNA}} = \frac{{{\text{DNA stimulated with LPS}} - {\text{digested DNA by DNase}} }}{{{\text{DNA treated with Triton}} \times 100}}.$$

### Disk diffusion assay

The effect of NETs against bacteria was tested according to the “EUCAST Disk diffusion method 6.0”. A disk *Diffusion Assay* was performed against *Staphylococcus aureus* NCTC 6571. The bacterial samples were plated in the Muller Hinton Agar medium and paper disks (6.0 mm diameter) soaked with 10 μl of NETs were placed on the plates and incubated at 37 °C for 24 h. NETs concentrations were normalized at 2 μg/ml. Inhibition halos diameter was measured in millimeter using a caliber.

### Keratinocyte proliferation

Both NETs isolated from adult and elderly volunteers were tested on human spontaneously immortalized keratinocyte (HaCaT) proliferation.

Neutrophils obtained by adult and elderly volunteers were seeded in 60 mm Petri dishes, and stimulated with 250 μg/ml LPS (Sigma Aldrich, Saint Louis, MO, USA). The neutrophils were incubated for 4 h in a humidified atmosphere containing 5% CO_2_ at 37 °C, then supernatant containing NETs was collected. NETs concentration was measured as previously reported [13].

HaCaT was incubated in cell culture medium without phenol red (Euroclone, Milan, Italy) to avoid colorimetric interference. HaCaT cells (10 × 10^3^ cells/well) were seeded into a 96 multi-well plate, treated with increasing NETs concentrations (0–500 ng/ml of DNA) and incubated for 48 h in a humidified atmosphere containing 5% CO_2_ at 37 °C. Upon the incubation, 0.5 mg/ml MTT (Thermo Fisher Scientific, Waltham, MA, USA) was added to wells for 3 h at 37 °C to allow formazan salts’ precipitation. The resulting insoluble purple precipitate was then dissolved in DMSO (Carlo Erba Reagents, Cornaredo, Italy) and the absorbance was read at 570 nm using a microplate reader (Victor X4, Perkin-Elmer, Waltham, MA, USA).

### NETs analysis by flow cytometry

To evaluate whether the different activities of NETs isolated from adult and elderly volunteers were due to their structural difference, NETs fragmentation was assessed by flow cytometry using a modification of the method of Delobel and Tesnière [21].

NETs obtained both from adult and elderly subjects (5 μg) were diluted in 2 ml PBS, incubated for 30 min at 37 °C with SYBR Green dye (10 μg/ml) and then analyzed using the flow cytometer (FACSCALIBUR, BD bioscience, San Jose, CA) equipped with a 15 mW air-cooled argon ion laser operating at 488 nm. Green fluorescence was collected through a 525 nm BP filter and fluorescence data were displayed on a four-decade logarithmic scale. A minimum of 10,000 events per sample were collected at a low sample flow rate setting (12 μl/min) to improve the coefficient of variation on the DNA histograms. Data were collected using the CellQuest software (BD Bioscience, San Jose, CA, USA), and then, analyzed using Flowing Software 2.5.2 (University of Turku, Finland).

### Statistical analysis

Statistical analysis was performed with the GraphPad statistical software (GraphPad Software Inc., CA, USA). The statistical significance of the data obtained was evaluated using the ANOVA-Bonferroni test. The *p* values < 0.05 was considered statistically significant.

## Results

### LPS induced a high NETs release in elderly subjects compared to adult ones

**Fig. 1:**
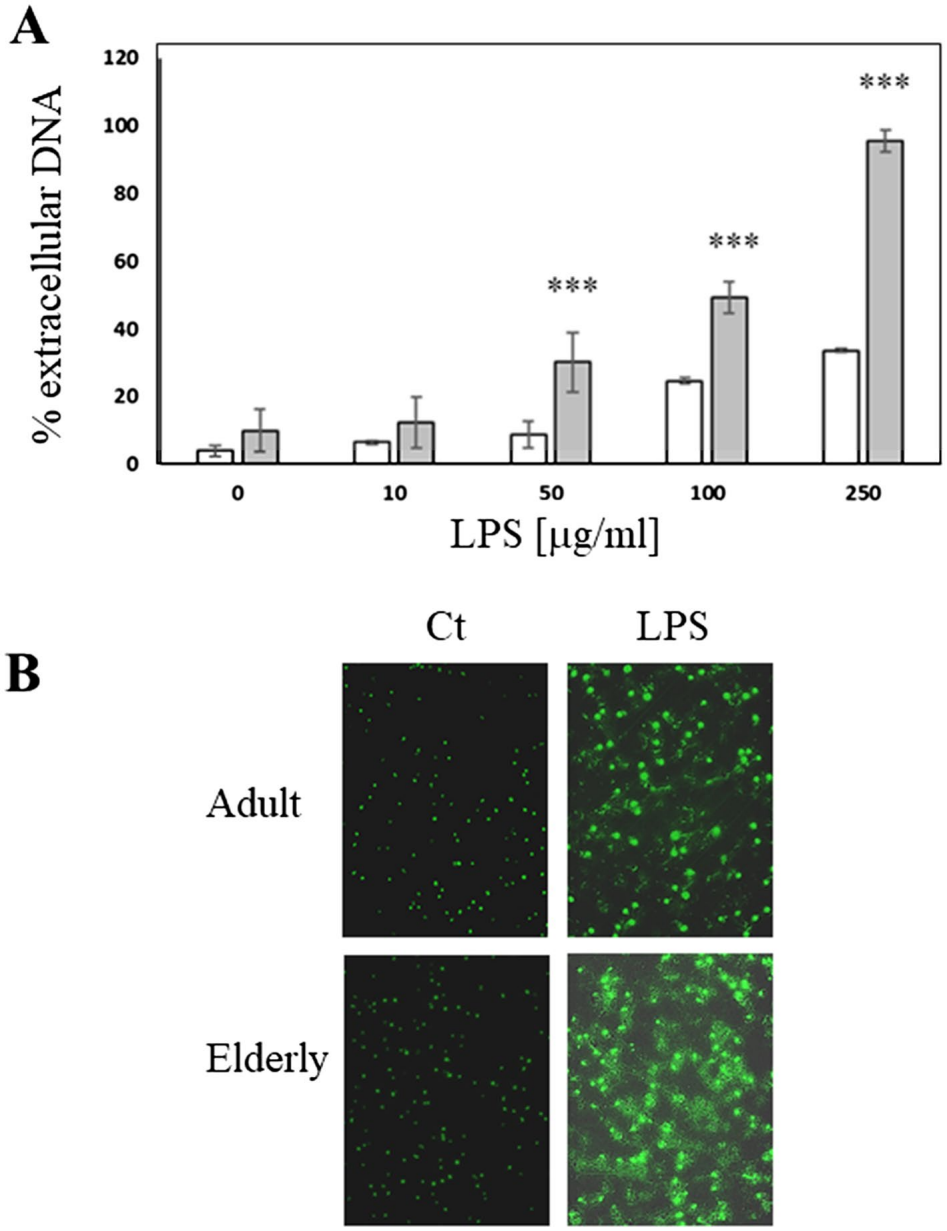
NETs released from human granulocytes after LPS stimulation. **A** Extracellular DNA quantification. LPS (0–250 μg/ml) was used to stimulate NETs production in 1 × 10^5^ neutrophils per well into a 96 multi-well culture plate. White bars = adult subjects, gray bars = elderly subjects ***p < 0.001 compared to adult; **B** representative fluorescence images of LPS-induced NETs production obtained after 10 μg/ml SYBR Green staining. Original magnification 40 ×. Ct = unstimulated granulocytes; LPS = granulocytes stimulated with 250 μg/ml LPS

Neutrophils extracted from two different groups of healthy volunteers (adult and elderly) were stimulated with LPS. Stimulation was performed using LPS (0–250 μg/ml) for 4 h (Fig. 1A). Both groups produced NETs in a dose-dependent manner, but elderly subjects generated a larger quantity of NETs than adults (Fig. 1A, B). This difference in the amount of NETs between the two groups became statistically significant starting from an LPS concentration of 50 μg/ml, when the cells from elderly volunteers released an amount of extracellular DNA equal to 30 ± 9% of total DNA present in the cell, against the 9 ± 4% produced by neutrophils from adult subjects. Furthermore, using 100 μg/ml LPS, NETs produced by the elderly were 50 ± 4.5% compared to 25% ± 0.9% of adults. Finally, stimulation with 250 μg/ml LPS induced the release of 95 ± 3% of the total DNA of elderly volunteers, which represents almost all the cellular DNA, whereas the adults released only the 33 ± 0.7% of the total DNA (Fig. 1A). Differences in 250 μg/ml LPS-induced NETs production in adult and elderly subjects were also evident in samples stained with SYBER Green dye and observed in fluorescence microscopy (Fig. 1B).

### NETs produced by neutrophils from elderly people displayed a lower antimicrobial activity compared to adult subjects

**Fig. 2:**
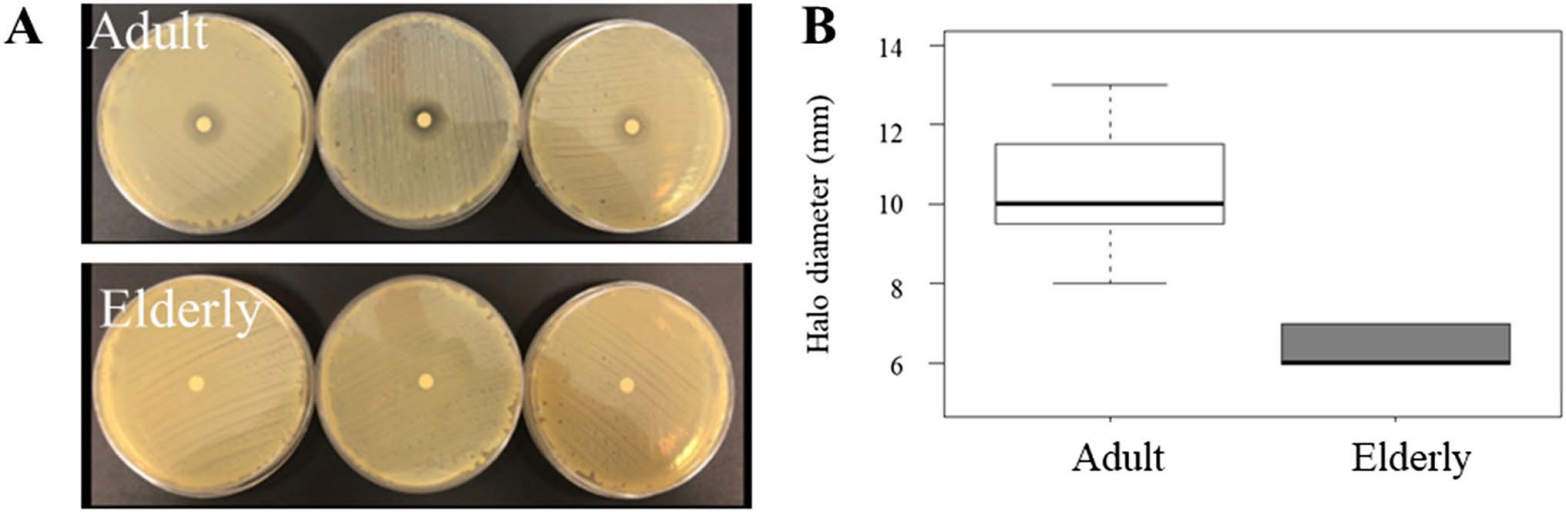
Evaluation of antimicrobial activity of NETs against *S. aureus.* **A** Representative images of inhibition growth halo induced in *S. aureus* by the NETs from adult and elderly subjects. **B** Quantification of halo dimensions induced by NETs from adult and elderly groups. ***p < 0.001

NETs antimicrobial activity was tested using a disk diffusion assay performed against *Staphylococcus aureus*. To test the effective microbicidal action of NETs, produced by both adult and elderly subjects, NETs concentrations were normalized at 2 μg/ml. Disks soaked using NETs from adults created an inhibition halo with a diameter equal to 10.33 ± 0.48 mm; while those soaked with NETs obtained from elderly people created a halo with a significantly smaller diameter (6.33 ± 0.14 mm *p* < 0.05) (Fig. 2A, B).

### HaCaT cells proliferate when stimulated only by NETs isolated from adult volunteers

NETs have been proved to induce proliferation of human keratinocytes via Toll-Like Receptor 9 (TLR 9) and NF-kB pathway activation [13]. To test the influence of NETs on wound healing process during aging, an MTT proliferation test on spontaneously immortalized human keratinocytes (HaCaT) was performed using different concentrations of NETs obtained from adult and elderly subjects.

**Fig. 3:**
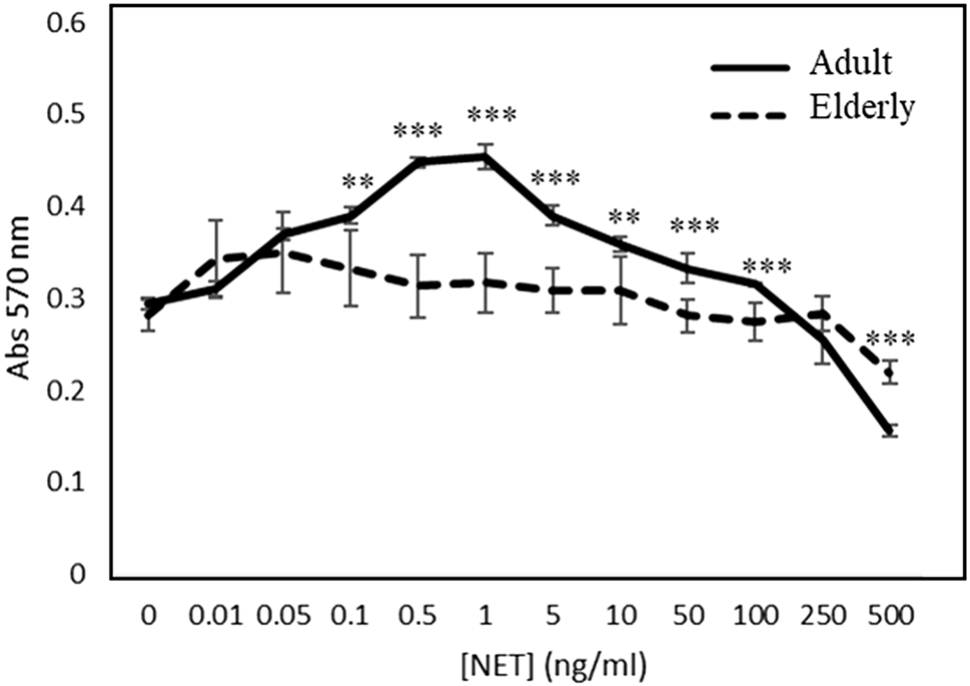
Human keratinocytes proliferation induced by NETs. MTT assay was to evaluate cell proliferation. MTT was performed on HaCaT cells stimulated with NETs (0–500 ng/ml) obtained by granulocytes from adult (continuous line) and elderly (dashed line) for 48 h. ***p < 0.001 referred to elderly subject

In the adult group, the proliferation of HaCaT cells was stimulated by NETs in a concentration range of 0.1–5 ng/ml; then, the proliferation slowed down from a concentration of 10 ng/ml, while over 100 ng/ml, a cytotoxic effect became evident (Fig. 3). On the contrary, in elderly group, no proliferative effect was observed at any NETs concentration (Fig. 3).

### Flow cytometric analysis showed a different fragmentation pattern in NETs from adult and elderly subjects

**Fig. 4:**
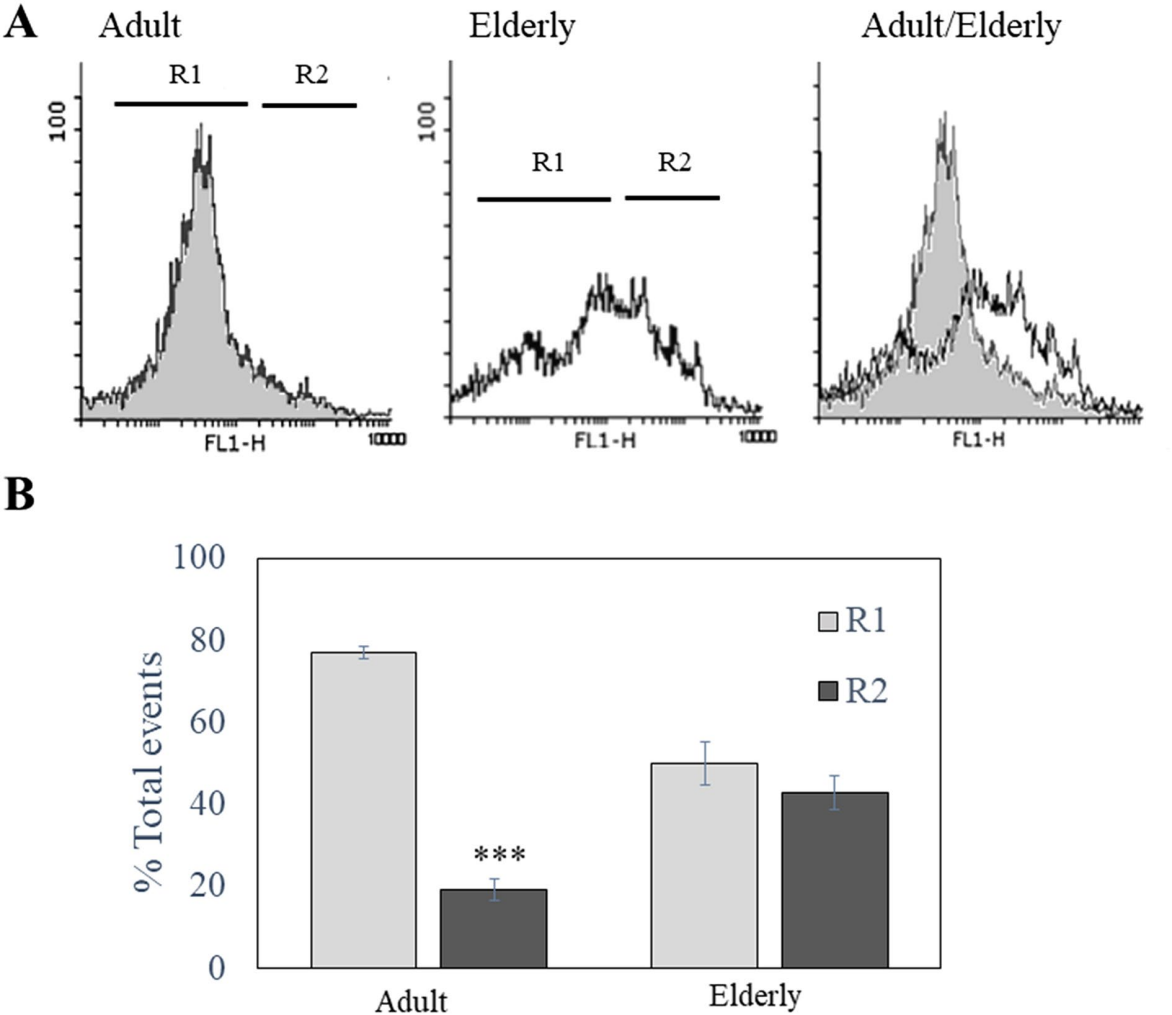
Flow cytometric analysis of NETs DNA size. **A** Representative fluorescence histograms relative to DNA presents in NETS obtained from adult and elderly subjects. DNA was staining with SYBR Green dye (10 μg/ml) and green fluorescence obtained was displayed in a logarithmic scale. Region R1 was created to include the majority (more than 70%) of fluorescence obtained in adult samples; in samples from elderly subjects, DNA showed a different fluorescence distribution with a population showing higher fluorescence and, therefore, higher size (collected in Region R2) compared to adult samples. To stress the shift in DNA size during the aging, an overlapped histogram was added (adult/elderly). **B** Quantification of fluorescence events distribution between R1 and R2 in adult and elderly groups expressed as % of total events. ***p < 0.001 referred to R1

A simple flow cytometric DNA fragmentation analysis was performed to assess whether the different antimicrobial and proliferative abilities observed in the NETs from adults and the elderly were due to a structural modification of the NETs. NETs were stained with SYBR Green and the green fluorescence obtained from the DNA-linked dye was read using a logarithmic scale (Fig. 4A). The DNA of NETs from adults appeared mainly distributed along a Gaussian-like curve with a low fluorescence intensity (Fig. 4A, Region 1), while the DNA of the elderly was distributed over two different populations: a “low intensity” population, formed by small sized DNA (Region 1, R1), and a “high intensity” population consisted of large DNA less fragmented (Region 2, R1). Quantitative analysis of the DNA distribution in R1 and R2 (Fig. 4B) showed that the 43.0 ± 4.1% of DNA from NETS obtained from elderly subjects was in R2 compared to the 19.3 ± 2.6% of the NETs from adult. Therefore, a real shift in the DNA size of the NETs during aging was observed (Fig. 4A).

## Discussion

During aging, the human immune system undergoes a process of decline in its protective function, making our body more susceptible to infections [22, 23]. This decline affects also neutrophils activities, and their immuno-senescence is rather related to a reduced efficiency in killing microbes than to a reduced number of circulating granulocytes or to a reduced efficiency of chemotaxis [15, 24–26]. Indeed, aging-related alterations observed in neutrophils appear to be related to altered signaling pathways, such as the JAK/Stat and PI3-K/Akt/ERK1/2, resulting in an impaired production of cytokines and ROS [27, 28].

The alteration of NETosis due to aging has been poorly investigated although with controversial results.

We observed that neutrophils from elderly subjects respond to LPS stimulation by producing a greater amount of NETs compared to neutrophils obtained from adult subjects.

This effect could be linked to an altered redox activity in granulocytes obtained from elderly subjects, as a similar effect was observed in aged mice. In fact, when granulocytes from aged mice were stimulated by 7-ketocholesterol, an athero-relevant stimulus showed an increased production of NETs linked to an increase in oxidative stress, caused by mitochondrial ROS production [29]. Therefore, mitochondria in neutrophils could act as powerful ROS generators to facilitate the innate immune function via NADPH-independent formation of NETs [30, 31].

Indeed, it has been reported that in aging, the key enzymatic activity responsible for NETs production, the Protein Arginine Deiminases-4 (PAD4), spontaneously generates NETs, and NETosis resulted exacerbated after stimulation [32, 33].

The observed increase in NETs production in elderly subjects is in contrast with the results obtained by Tseng et al. [16], who, in a murine model of *Staphylococcus* *aureus* infection, observed lower NETs production in neutrophils obtained by elderly animals compared to young ones. Moreover, Hazeldine et al. [17] observed in TNFα-primed human PMN a reduction of NETs production in aging. A possible explanation for this discrepancy could be due to both the LPS concentrations used for NETs production (10 ng/ml) and cells’ stimulation condition used (TNF-α primed human neutrophils) [15, 22, 27]. Neutrophils from the elderly have shown a pre-activated baseline state with increased ROS production compared to neutrophils from young subjects [34, 35]. This may account for the differences observed between activated and resting neutrophils [36] and explain why aged-primed neutrophils produced a lower quantity of NETs than young ones, while aged not-primed neutrophils produce more NETs than young ones.

Tseng et al. [16] stimulated murine PMN directly with *S. aureus* and PMA (20 nM). Although PMA has been extensively used to study the biology of NETs in many publications, it is not a physiological stimulus [37], and it does not activate the JNK-dependent pathway [18].

Moreover, it could be interesting to compare in our experimental model the different quantification methods of NETs used in the literature to clarify the difference in NETs production in healthy elderly and adult subjects. However, despite increased NETs production in elderly subjects, these NETs showed less antimicrobial activity against *S. aureus*.

NETs antimicrobial activity is due both to the presence of high concentrations of antimicrobial molecules linked to the DNA meshwork and DNA ability to chelate manganese (Mn^2+^) and other divalent cations (Ca^2+^, Mg^2+^). Mn^2+^ acts as an electron transporter on the bacterial wall, playing an important role in microbial survival and proliferation, and its chelation prevents this transport [6, 7, 38]. DNA chelating activity is linked to its thread length, as long DNA fragments showed low chelating activity and bactericidal effect [39]. Even very small fragments of DNA showed a low bactericidal effect, as bacteria extracellular DNase activity facilitates escape from NETs [40].

We analyzed soluble NETs in flow cytometry and observed that DNA from elderly subjects exhibited a shift towards larger fragments compared to DNA size distribution observed in adult subjects.

The presence of large DNA fragments in NETs could justify the low bactericidal activity observed.

We have previously demonstrated that NETs induce proliferation in human keratinocytes (HaCaT) [13], NETs act on keratinocytes through the internalization of double-strand DNA by TLR9 receptors, which induce a NF-kB-dependent proliferation of keratinocytes [16]. This phenomenon is NET-concentration-dependent, with low physiological NETs concentration increasing keratinocyte proliferation, while high NETs concentration reduces their proliferation [13]. In our experiments, NETs from elderly failed to stimulate cell proliferation.

In addition, in this case, large DNA fragments have been found to be less efficient in the interaction with and activation of TLR9 [39]. The presence of large (or perhaps less decondensed) DNA fragments in NETs could be due to the epigenetic structure of the DNA such as age-related DNA methylation patterns [41, 42]. Other possible epigenetic mechanism is change in chromatin structure so that genomic DNA is wrapped within a chromatin structure that both compacts and protects DNA from interaction with surrounding factors. The basic unit of chromatin (nucleosome) packages the DNA with histone proteins such as H3 and H4. Methylated DNA gets wrapped around nucleosomes in a tight conformation which makes it relatively inaccessible [41, 42]. Therefore, some methylated chromatin areas could produce large fragments of NETs.

This hypothesis may explain our results, being larger DNA threads less effective in inducing both microbial toxicity and keratinocyte proliferation.

The presence of large size of DNA threads in NETs from elderly subjects has not been previously reported and it needs a more careful analysis. In fact, NETs degradation is found to be impaired in several autoimmune diseases, such as SLE [43–45] mainly due to DNase I deficiency, and compromised NET clearance in turn leads to inflammation and NETs autoantigens production [46]. Large size DNA fragments in NETs could be more difficult to clear becoming a trigger for an increased inflammation and autoimmune response in aging. Therefore, since NETs contain various active enzymes and DAMPs [47], their physiological clearance by DNase 1 action and macrophage phagocytosis is crucial [43, 48].

In conclusion, our results show a less explored aspect of the NETs linked to aging, consisting in a modification of their structure, resulting in both less antimicrobial activity and stimulation of keratinocyte proliferation, compared to NETs obtained from adult subjects. Both effects could have a major impact on wound healing process in healthy elderly subjects.
